# Editorial: Engaging scientific diasporas for development: Policy and practices

**DOI:** 10.3389/frma.2022.1102805

**Published:** 2023-01-04

**Authors:** Kleinsy Bonilla, Luisa F. Echeverría-King, Milena Serafim, Tebogo Mabotha, Derya Buyuktanir Karacan

**Affiliations:** ^1^Department of Science and Technology Policy, State University of Campinas, Campinas, Brazil; ^2^Instituto para el Desarrollo de la Educación Superior en Guatemala (INDESGUA), Guatemala, Guatemala; ^3^Faculty of Applied Sciences FCA, UNICAMP, Corporación Universitaria del Caribe, Sincelejo, Colombia; ^4^Office of Strategic Partnerships at the Academy of Science of South Africa (ASSAf), Pretoria, South Africa; ^5^Elliott School of International Affairs, George Washington University, Washington, DC, United States

**Keywords:** scientific diasporas, global south, migration and development, brain drain and brain gain, science diasporas, science technology and innovation (STI), S&T policy, science diplomacy

The study of scientific diasporas (SD) is more relevant than ever, and so is having a shift in the paradigm. The emigration of highly educated and science-skilled individuals from countries in the South to countries in the North has predominantly been understood as a loss of scientific knowledge and capabilities by the countries of origin for the benefit of the countries of destination. This has been known in the literature as “brain drain” and has remained a widely used paradigm for decades. However, a shift in such a conceptual framework has emerged, considering that such SD can turn into a valuable bridge between scientifically lagging and scientifically advanced nations.

Various countries are increasingly building connections between their national needs and interests with the capabilities of their SD. Such nations aim at strengthening linkages with their SD in the face of alliances for sustainable development. In addition, the SD from the Global South play an active role in the construction of academic networks and partnerships for transnational science projects. SD can support and enhance the quality of higher education in their home country, by bridging low levels of public and private investment through their capacities, learned technologies, and high-level training. On the other hand, the SD's identification, mapping, and characterization remain challenging for most developing regions of the world. Today's increasingly interconnected and globalized societies require the active circulation of knowledge and technology among nations. For this, it is necessary that policymakers, particularly those related to foreign service, generate effective and sustainable engagement initiatives with their SD. Various stakeholders are relevant, including universities, science academies, corporate firms, and international organizations.

This Research Topic came about as an attempt to address the limited literature on the highly qualified diaspora and their mobilization to different countries, as well as the lack of information on good practices and public policy strategies for engaging the SD. This Research Topic, therefore, seeks to present not only empirical research conducted in various parts of the world but also to include representative cases and policy analysis. The papers published as part of this Research Topic show the plurality of edges the engagement of SD can entail and portray the multiple challenges faced by countries of origin to recognize, identify and map their SD as a critical first step to later engage with such SD for the development of their territories. These 14 papers are divided into three groups.

The first group of five papers seeks to contribute to the debate on the scientific diaspora and its potential to contribute to science diplomacy initiatives. The article “*Emerging Technologies, STI Diaspora and Science Diplomacy in India: Towards a New Approach*” (Pandey et al.) examines India's initiatives and strategies for engagement with the scientific diaspora. Using examples from other countries, this study outlines an approach with some examples of strategies and initiatives for harnessing science diplomacy to enhance engagement with the SD to create a win-win milieu for India and the diaspora. The paper “*Organized Scientific Diaspora and its Contributions to Science Diplomacy in Emerging Economies: The Case of Latin America and the Caribbean*” (Echeverria-King et al.) analyzes the interactions and initiatives identified between the organized scientific diaspora from Latin America and the Caribbean and their countries of origin concerning science diplomacy processes, providing recommendations and proposals for public policy to improve the interaction between the diaspora and the governments of their countries of origin. The opinion paper “*The Potential Contribution of the Scientific Diaspora to Enhance Marine Science in Guatemala*” (Barrios-Guzman and de la Cruz) is in line with the previous article, in the sense that it highlights the potential of the scientific diaspora and how it would contribute to the strengthening of science diplomacy. Based on a specific case study, the authors understand that academic and scientific actors outside the country could be a bridge for the execution of cooperation projects and activities, facilitating the exchange and transfer of knowledge and technology. Complementary to this, the paper “*Recognize and Alleviate a Resource Management Conundrum Facing Science Diaspora Networks*” (Butler et al.) contributes to the debate on science diaspora networks, understanding networked organizations and how they manage resources. The work provides a new database of operational science diaspora networks. The article “*Scientific Diasporas and the Advancement of Science Diplomacy: The InFEWS US-China Program in the Face of Confrontational ‘America First' Diplomacy*,” (Prieto and Scott) presents a descriptive analysis of how science diplomacy played a critical role between the United States and China through the Innovations at the Nexus of Food, Energy, and Water Systems (InFEWS) US-China program, by promoting scientific collaborations to expand the food, energy and water nexus research and applications.

The second group composed of six papers, explored case studies of SD engagement in different countries, identifying and analyzing the peculiarities of each experience. The Guatemalan experience was studied in three papers. The paper “*Engaging the Guatemala Scientific Diaspora: The Power of Networking and Shared Learning*” (Bonilla et al.) outlines the Guatemalan scientific diasporas' (GSD) networking as a mechanism for building research excellence and intellectual capital. Findings highlight the importance of digital and technological pathways that might leverage the GSDs' knowledge and experience, channeling skills, and international connections for better interaction with Guatemalan society. Complementarily, the paper “*Connecting Scientists Residing Abroad: A Review of Converciencia as a Practice to Engage the Guatemalan Scientific Diaspora From 2005–2020*” (Bonilla, Arrechea et al.) analyzes the *Converciencia* program, designed to connect Guatemalan scientists residing abroad with their country of origin. This article presents a comprehensive and balanced overview of the program applying an in-depth analysis of its creation, evolution, leading trends, and legacies. The paper “*Developing a Digital Technology System to Address COVID-19 Health Needs in Guatemala: A Scientific Diaspora Case Study*” (Alvarado et al.) shows that SD are organized groups of professionals who can work together to contribute to their country of origin. Their collaboration was facilitated by FUNDEGUA, a Guatemalan non-profit, which provided a legal framework to establish partnerships and raise funds. The paper analyzed an initiative known as ALMA (*Asistente de Log*í*stica Médica Automatizada*), showing how SD can provide an avenue for professionals to contribute to Guatemala, regardless of their residence and job commitments. The Colombian experience outlined in the paper, “*Engaging Scientific Diasporas in STEAM Education: The Case of Science Clubs Colombia*,” (Avendano-Uribe et al.) showcases the Science Clubs Colombia (*Clubes de Ciencia Colombia*-SCC) program, a pioneering STEAM capacity-building initiative led by volunteer scientists to engage youth and children from underserved communities in science. The program brings together researchers based in Colombia and abroad to lead intensive project-based learning workshops for young students in urban and rural areas. In the Honduras experience, the paper, “*Engaging Honduran Science Diasporas for Development: Evidence from Three Consolidated Networks*,” (Bonilla, Valle et al.) portrays the dynamics of three consolidated diaspora networks, which provide evidence of their existence and engagement in their home country. Evidence showed that the Honduran SD are willing to transfer knowledge, build bridges, facilitate access to world-class research practices to their peers residing in Honduras while interacting with the broader sectors of the Honduran society. The Mexican experience is presented in the article “*Mexican Scientist Diaspora in North America: a perspective on collaborations with México*” (Gómez-Flores et al.) explored how the Mexican SD residing in North America are committed to aiding the country's development. However, a lack of institutional coordination has not harnessed their contribution to capacity building.

Finally, the last group of three articles delves into the relationship between indicators and the level of engagement of the SD. The article “*RAICEX: A Successful Story of the Spanish Scientific Diaspora*” (Ortega-Paino and Oliver) analyzed the role of the Network of Associations of Spanish Researchers and Scientists Abroad and surveyed the experiences of Spanish researchers distributed in several countries on five different continents. The main objectives of the program were to provide support to researchers and scientists in mobility and personal development, to disseminate and give visibility to the value of science and the work of researchers and scientists, promote communication of the advances of knowledge in all areas of society and promote international relations and cooperation. In the paper “*Biodiversity Research in Central America: A Regional Comparison in Scientific Production Using Bibliometrics and Democracy Indicators*,” (Morales-Marroquin et al.) the aim was to show how the democratic shifts throughout the years have impacted the science production on biodiversity research and species records. The paper discusses democracy, science production, funding, and conservation as core elements that go hand in hand and that need to be nourished in a region that struggles with protecting life and extractive activities in a climate change scenario. Closing the collection, the article “*Voices of the Costa Rican Scientific Diaspora: Policy Lessons from a Decade of Experiences from our Scientists Abroad*” (Jarquin-Solis et al.) analyzes 10 years of diaspora perspectives as reflected by those interviewed. Authors extracted insights about the most common mechanisms and funding sources used by Costa Rican scientists seeking professional opportunities overseas, their level of engagement and collaboration with Costa Rican research and higher education institutions, and the possible incentives that the country could prioritize to harness their scientific talent and increase scientific capacities at home.

We, the co-editors, are convinced that these articles can contribute to future efforts and strategies for the engagement of SD from the Global South in the development processes of their country of origin. We believe that a major contribution of the Research Topic is the inclusion of different contexts and geographies. In addition, this Research Topic contributes to increasing the visibility of the engagement of SD for development, allowing for counterbalance on the emphasis, focus, and narratives that have dominated the literature. We look forward to seeing a growing interest in studying and understanding the dynamics of SD from the perspective of their countries of origin. We thank all the authors, co-authors, reviewers, and external editors who participated in this process. We also thank Frontiers in Research Metrics and Analytics for making it possible to publish this Research Topic and all the articles as part of this collection.

## Author contributions

KB: conceptualization (lead), structure (lead), methodology (lead), resources (lead), validation (lead), and writing—original draft (lead). LE-K and MS: conceptualization (equal) and writing (equal). TM: copyediting (lead). DB: contribution of ideas (assisting). All authors contributed to the article and approved the submitted version.

